# Chronic Tinnitus Exhibits Bidirectional Functional Dysconnectivity in Frontostriatal Circuit

**DOI:** 10.3389/fnins.2019.01299

**Published:** 2019-12-06

**Authors:** Jin-Jing Xu, Jinluan Cui, Yuan Feng, Wei Yong, Huiyou Chen, Yu-Chen Chen, Xindao Yin, Yuanqing Wu

**Affiliations:** ^1^Department of Otolaryngology, Nanjing First Hospital, Nanjing Medical University, Nanjing, China; ^2^Department of Radiology, Nanjing First Hospital, Nanjing Medical University, Nanjing, China

**Keywords:** tinnitus, nucleus accumbens, prefrontal cortex, directional connectivity, resting-state fMRI

## Abstract

**Purpose:**

The phantom sound of tinnitus is considered to be associated with abnormal functional coupling between the nucleus accumbens (NAc) and the prefrontal cortex, which may form a frontostriatal top-down gating system to evaluate and modulate sensory signals. Resting-state functional magnetic resonance imaging (fMRI) was used to recognize the aberrant directional connectivity of the NAc in chronic tinnitus and to ascertain the relationship between this connectivity and tinnitus characteristics.

**Methods:**

Participants included chronic tinnitus patients (*n* = 50) and healthy controls (*n* = 55), matched for age, sex, education, and hearing thresholds. The hearing status of both groups was comparable. On the basis of the NAc as a seed region, a Granger causality analysis (GCA) study was conducted to investigate the directional connectivity and the relationship with tinnitus duration or distress.

**Results:**

Compared with healthy controls, tinnitus patients exhibited abnormal directional connectivity between the NAc and the prefrontal cortex, principally the middle frontal gyrus (MFG), orbitofrontal cortex (OFC), and inferior frontal gyrus (IFG). Additionally, positive correlations between tinnitus handicap questionnaire (THQ) scores and increased directional connectivity from the right NAc to the left MFG (*r* = 0.357, *p* = 0.015) and from the right MFG to the left NAc (*r* = 0.626, *p* < 0.001) were observed. Furthermore, the enhanced directional connectivity from the right NAc to the right OFC was positively associated with the duration of tinnitus (*r* = 0.599, *p* < 0.001).

**Conclusion:**

In concurrence with expectations, tinnitus distress was correlated with enhanced directional connectivity between the NAc and the prefrontal cortex. The current study not only helps illuminate the neural basis of the frontostriatal gating control of tinnitus sensation but also contributes to deciphering the neuropathological features of tinnitus.

## Introduction

Tinnitus is defined as a phantom auditory perception that may bother many patients to the extent that it changes their lives (Baguley et al., 2013; Bauer, 2018). Because of the aging population and unavoidable noise pollution, the incidence of chronic tinnitus has increased sharply over the past 15 years (Shargorodsky et al., 2010; Knipper et al., 2013). Currently, the mechanism of action of chronic and irreversible tinnitus is unknown. Hence, many investigators have applied themselves to unraveling the nature of tinnitus (Galazyuk et al., 2012), notably subjective chronic tinnitus without organic lesions. Abnormal cochlear function has been considered to be the primary cause of tinnitus over the years. However, a small number of studies indicated that many patients failed to experience relief from tinnitus after the complete recovery of cochlear lesions (Rauschecker et al., 2010). The central nervous system is integral when considering the occurrence and development of tinnitus. A series of anomalies in the auditory center were observed in tinnitus cases in prior research studies (Leaver et al., 2011, 2016a; Kim et al., 2012; Song et al., 2012, 2015; Vanneste and De Ridder, 2012; Adjamian et al., 2014; Husain and Schmidt, 2014; Chen et al., 2015, 2016; Hinkley et al., 2015; Gunbey et al., 2017; Schmidt et al., 2017; Hullfish et al., 2019), simultaneously covering synaptic remodeling (Eggermont and Roberts, 2004), neuronal spontaneous hyperactivity or hypoactivity (Chen et al., 2014), increased neuronal synchronicity (Eggermont, 2007; Langers et al., 2012), and changes in tonotopic representation of sound (Langers et al., 2012). Tinnitus activations may also involve the non-auditory center, in which the limbic system and the autonomic nervous system are especially important (Crippaa et al., 2010; Rauschecker et al., 2010; Leaver et al., 2011, 2016a; Kim et al., 2012; Vanneste and De Ridder, 2012; Carpenter-Thompson et al., 2014; Kreuzer et al., 2015; Song et al., 2015; Chen et al., 2016; Gunbey et al., 2017; Schmidt et al., 2017; Hullfish et al., 2019). As a result, an increasing number of researchers have investigated tinnitus from the periphery to the central nervous system. Although many studies have investigated this area over the years, the neuropathological mechanism underlying tinnitus generation remains an enigma.

Comprehensively accumulated evidence from functional neuroimaging studies indicated that the limbic system plays an intermediary role in evaluating and modulating tinnitus perception signals, for instance, identifying and eliminating phantom sounds normally as well as canceling the noise at the level of the limbic system by providing feedback to the thalamic reticular nucleus (TRN) (Muhlau et al., 2006; Rauschecker et al., 2010, 2015; Leaver et al., 2011; Barry et al., 2015; Gunbey et al., 2017). At the level of the thalamus, the inhibition and stimulation of the medial geniculate nucleus (MGN) and TRN are determined by the pathway above (Rauschecker et al., 2010, 2015; Barry et al., 2015). Research has also indicated that one of the reasons for the generation and maintenance of tinnitus is the breakdown of auditory circuitry, especially in the thalamic regions, including the MGN and TRN (Rauschecker et al., 2010; Barry et al., 2015). Moreover, the functional connectivity between the nucleus accumbens (NAc) and MGN single neurons as well as the modulation of auditory neuron activity in the MGN by the NAc may play a part in the development of tinnitus (Barry et al., 2015).

Furthermore, the generation of tinnitus may be ascribed to the reaction of the NAc to dysfunctional sensory gating control (Barry et al., 2015). The study of Rauschecker et al. demonstrated that as part of a central gatekeeping system in tinnitus, the subcallosal area, which is composed of the ventromedial prefrontal cortex (vmPFC) and the NAc, evaluates the relevance and emotional value of sensory stimuli, and controls the flow of information (Rauschecker et al., 2010). Previous studies have suggested that the NAc and vmPFC and subgenual anterior cingulate cortex (sgACC) form a frontostriatal gating circuit in tinnitus patients (Leaver et al., 2011, 2016b; Rauschecker et al., 2015; Hullfish et al., 2019). Therefore, functional magnetic resonance imaging (fMRI) was used to measure the neural activity within frontostriatal network in tinnitus.

The interconnection within cerebral networks, which is structurally segregated and functionally specialized, may be further understood by resting-state low-frequency (0.01–0.1 Hz) fluctuations of blood oxygenation level-dependent (BOLD) fMRI (Biswal et al., 1995; Raichle et al., 2000; Barkhof et al., 2014). Recent MRI studies on tinnitus have raised awareness of altered structural and functional brain alterations in auditory and limbic regions (Leaver et al., 2011, 2016a; Vanneste and De Ridder, 2012; Meyer et al., 2016; Hullfish et al., 2019). The NAc, vmPFC, and sgACC could be the kernel regions of tinnitus dysfunction. The highest degree of hyperactivity, specifically to sounds frequency matched in the NAc area, as well as complementary structural differences in the vmPFC, has been shown in tinnitus patients (Leaver et al., 2016a). Increased gray matter (GM) and decreased white matter (WM) concentrations in the vmPFC within tinnitus patients have been detected (Middleton and Tzounopoulos, 2012), in which the functional changes in the NAc and auditory cortex were closely associated with the degree of structural changes. Moreover, Meyer et al. (2016) demonstrated that the reduced GM in sgACC was correlated with tinnitus duration in a large sample of tinnitus patients, suggesting that ongoing tinnitus may be related to progressive reorganization of the sgACC, which is consistent with the gating model (Rauschecker et al., 2015). Likewise, Hinkley et al. (2015) investigated the presence of increased functional connectivity in the NAc and vmPFC with the auditory cortex of tinnitus patients. While many studies have shown that tinnitus involves changes in the functional connectivity between the whole brain and the NAc as well as the vmPFC (Leaver et al., 2011; Adjamian et al., 2014; Hullfish et al., 2019), the directionality or specificity of the dysfunctional connections in tinnitus remains unknown.

A Granger causality analysis (GCA) study is employed to identify directionality in altered effective connectivity between the whole brain and the NAc as well as the vmPFC among subjects, providing scientific evidence for in-depth research. GCA has been applied broadly to expose the causal effects among brain regions in other neurological or psychiatric disorders, such as Alzheimer’s disease (Zhong et al., 2014), mild cognitive impairment (Yu et al., 2017), depression (Guo et al., 2015), and presbycusis (Chen et al., 2019). In the current study, fMRI combined with the GCA method was used to collect and process BOLD data to precisely determine the directionality of altered resting-state directional connectivity (Jiao et al., 2011; Stephan and Roebroeck, 2012). Specific seed regions are stipulated in the NAc considering the potential role of the NAc within the frontostriatal gating circuit in tinnitus. It is hypothesized that disruption of the directional connectivity in the NAc in patients with chronic tinnitus would be observed by utilizing the GCA method. Moreover, the disrupted frontostriatal connectivity would be associated with specific tinnitus characteristics, such as tinnitus distress and duration. To our knowledge, this is the first study to use fMRI in combination with the GCA method to detect the directional connectivity of the NAc in chronic tinnitus patients.

## Materials and Methods

### Subjects

**FIGURE 1:**
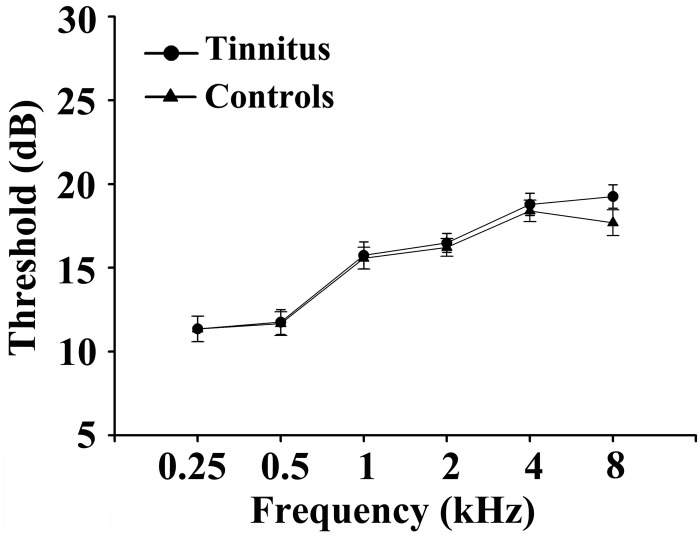
Mean hearing thresholds of the chronic tinnitus and control groups. Data are presented as mean ± SD.

Fifty chronic tinnitus patients and 55 healthy subjects (all right-handed, with at least 8 years of education) were recruited through community health screening or newspaper advertisements. No subject was excluded from the fMRI analysis due to excessive head motion during scanning. The patients and controls were paired into matched groups in terms of age, sex, and education. There were 18 left-sided and 16 right-sided tinnitus patients as well as 16 tinnitus patients who experienced bilateral tinnitus or tinnitus originating within the head. The Iowa version of the Tinnitus Handicap Questionnaires (THQ) (Kuk et al., 1990) and a pure tone audiometry (PTA) examination were used to assess the tinnitus severity, tinnitus distress, and the hearing threshold. All participants had clinically normal hearing (hearing thresholds < 25 dB) at the frequencies of 0.25, 0.5, 1, 2, 4, and 8 kHz. There were no statistically significant differences in auditory thresholds between the tinnitus group and the control group (see Figure 1 for average hearing thresholds). According to the self-rating depression scale (SDS) and the self-rating anxiety scale (SAS) (overall scores < 50, respectively), none of the participants had depression or anxiety (Zung, 1971, 1986). In accordance with a previous study (Khalfa et al., 2002), the Hyperacusis Questionnaire was applied to exclude participants with hyperacusis in the current study. Moreover, consistent with the previous diagnostic criteria (Lopez-Escamez et al., 2015), patients with Meniere’s disease were also excluded from the study. Other exclusion criteria for the study included the following: (1) pulsatile tinnitus, hyperacusis, or Meniere’s diseases; (2) a past history of severe alcoholism, smoking, or head injury; (3) stroke, Alzheimer’s disease, Parkinson’s disease, epilepsy, major depression, or other neurological or psychiatric illness; (4) major medical illness (e.g., cancer, anemia, and thyroid dysfunction); (5) severe visual loss; and (6) any contraindications for MRI scanning. The characteristics of the chronic tinnitus patients and healthy subjects are summarized in Table 1. The study was approved by the Ethics Committee of Nanjing Medical University, and informed consent was obtained from each participant.

**TABLE 1:** Demographic and clinical characteristics of chronic tinnitus patients and healthy controls.

	**Tinnitus patients (*n* = 50)**	**Healthy controls (*n* = 55)**	***p*-value**
Age (year)	50.20 ± 11.19	46.82 ± 11.99	0.139
Gender (male: female)	18:32	21:34	0.817
Education levels (years)	12.50 ± 2.93	12.674 ± 3.04	0.768
Tinnitus duration (months)	37.71 ± 34.58	–	–
THQ score	52.22 ± 15.08	–	–
Hearing thresholds (left)	16.79 ± 2.76	16.87 ± 2.55	0.890
Hearing thresholds (right)	16.60 ± 3.37	16.95 ± 2.35	0.569
Hearing thresholds (average)	16.69 ± 2.68	16.91 ± 1.62	0.642

*Data are represented as mean ± SD. The PTA from both ears was averaged. PTA, pure tone audiometry; THQ, tinnitus handicap questionnaires.*

### MRI Scanning

A 3.0-T MRI scanner (Ingenia, Philips Medical Systems, Netherlands) with an eight-channel receiver array head coil was used to generate magnetic resonance images. Foam padding and earplugs were used to reduce the head motion and scanner noise. From the manufacturer’s data, approximately 32 dB of scanner noise may be attenuated by the earplugs (Hearos Ultimate Softness Series, United States). All participants were instructed to remain awake, keep their eyes closed, and stay motionless without thinking of anything in particular during scanning. Structural T1-weighted images were acquired in a high-resolution three-dimensional turbo fast echo (3D-TFE) scan with the following parameters: repetition time (TR)/echo time (TE) = 8.1/3.7 ms; slices = 170; thickness = 1 mm; gap = 0 mm; flip angle (FA) = 8°; acquisition matrix = 256 × 256; and field of view (FOV) = 256 mm × 256 mm. A total of 5 min 29 s was spent in scanning the structural sequence. Functional images were collected axially using a gradient-recalled echo-planar imaging sequence as follows: TR = 2,000 ms; TE = 30 ms; slices = 36; thickness = 4 mm; gap = 0 mm; FOV = 240 mm × 240 mm; acquisition matrix = 64 × 64; and FA = 90°. A total of 8 min 8 s was spent on the fMRI sequence.

### Data Preprocessing

Data Processing & Analysis for Resting-State Brain Imaging (DPABI_V2.3_170105) with the following stages was applied for data analysis (Yan et al., 2016). The first 10 volumes were discarded to reduce deviation, and the remaining 230 consecutive volumes were used for data analysis. Slice timing and realignment for head motion correction were performed. Subjects were excluded if they exhibited a head motion with >2.0-mm translation or a 2.0° rotation in any direction. The Montreal Neurological Institute (MNI) template (resampling voxel size = 3 mm × 3 mm × 3 mm) and an isotropic Gaussian kernel [full width at half maximum (FWHM) = 6 mm] were employed to spatially normalize, detrend, and filter (0.01–0.08 Hz) the data.

With the WFU_PickAtlas software^1^, the bilateral NAc were set as seed regions. Moreover, REST-GCA in the REST toolbox was applied to analyze the effective connectivity (Zang et al., 2012). The current study established the seed time series *x* by the time series of the bilateral NAc and the time series *y* by the time series of all voxels in the brain. The linear direct influence of *x* on *y* (*F_x→y_*) and of *y* on *x* (*F_y→x_*) was calculated on the basis of the voxel across the brain. Thus, two Granger causality maps were generated according to the influence measures for each subject. The residual-based *F* was normalized (*F*’) and standardized to the *Z* score for each voxel (*Z_x→y_* and *Z_y→x_*, subtracting the global mean *F*’ values, divided by the standard deviation).

### Structural Analysis

Structural images were processed using the VBM8 toolbox software in SPM8^2^. Imaging preprocessing was performed in accordance with the optimized VBM protocol previously described by Good et al. (2001) including spatial normalization, segmentation, modulation, and smoothing. In SPM8, the image is rearranged to the front and back joint axis. After rearrangement, the images were divided into GM, WM, and cerebrospinal fluid using the full-automatic algorithm in VBM8. The segmented images were used to create a custom DARTEL template, which was then normalized to the MNI space. The resulting GM and WM images were smoothed using a 10-mm FWHM Gaussian kernel. GM and WM volumes were calculated by estimating these segments. Brain parenchyma volume was calculated as the sum of GM and WM volumes. The voxel-wise GM volume was used in the following statistical analysis as covariates for GCA calculations.

### Statistical Analysis

The mean values of the *Z_x→y_* and *Z_y→x_* maps were computed several times to analyze the effective connectivity of the bilateral NAc between groups. On the basis of the above results, four Granger causality maps, including two for each direction and two for each group (the left NAc with *Z_x→y_* and *Z_y→x_* and the right NAc with *Z_x→y_* and *Z_y→x_* for both the patients and healthy controls), were acquired. Thus, to determine the differences between tinnitus patients and healthy controls, in which the covariates were age, sex, and education, these Granger causality maps were entered into a voxel-wise two-sample *t*-test. To exclude potential effects of GM volume differences, the voxel-wise GM volume maps were also obtained as covariates. The voxel-wise results for group differences were corrected for multiple comparisons using 3dClustSim^3^ determined by Monte Carlo simulation. A combined threshold of *p* < 0.001 and a minimum cluster size of 40 voxels were set, yielding a corrected threshold of *p* < 0.01.

Demographic data were compared by using between-group *t*-tests and χ^2^ tests (statistical significance set at *p* < 0.05). The clusters of the significant differences in the effective connectivity of the bilateral NAc between groups were extracted to investigate the association between the clinical characteristics and the fMRI data. Partial correlations in SPSS software (version 19.0; SPSS, Chicago, IL, United States) were used to correlate the mean *z* values within these clusters with the characteristics of each tinnitus patient. After correction for age, sex, education, and hearing thresholds, *p* < 0.05 was considered statistically significant. Bonferroni correction for multiple comparisons was applied in the correlation analysis.

## Results

### Structural Data

After VBM analysis, there were no significant differences in the comparisons of the whole-brain volumes (GM volume, WM volume, and brain parenchyma volume) between chronic tinnitus patients and healthy controls (*p* > 0.05). After Monte Carlo simulation correction, no suprathreshold voxel-wise difference in the GM and WM volume between the chronic tinnitus patients and healthy controls was observed.

### Directional Connectivity From the NAc

**FIGURE 2:**
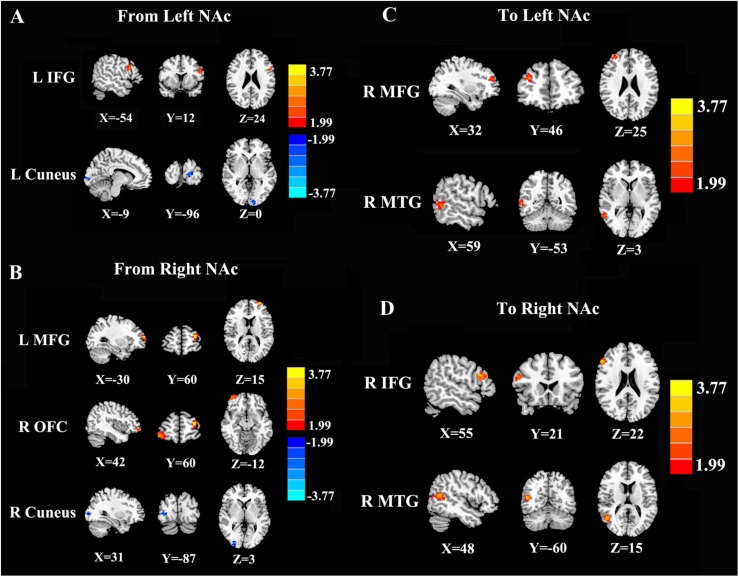
Directional functional connectivity of the bilateral NAc in chronic tinnitus patients compared with healthy controls (*p* < 0.01 corrected by 3dClustSim). **(A)** Reduced directional connectivity from the left NAc to the left IFG and left cuneus. **(B)** Decreased directional connectivity from the right NAc to the left MFG, right OFC, and right cuneus. **(C)** Reduced directional connectivity from the right MFG and right MTG to the left NAc. **(D)** Decreased directional connectivity from the right IFG and right MTG to the right NAc.

In contrast to healthy controls, patients with chronic tinnitus demonstrated significantly increased directional connectivity from the left NAc to the left inferior frontal gyrus (IFG). Interestingly, decreased directional connectivity was detected in the left cuneus. In addition, significantly enhanced directional connectivity from the right NAc to the left middle frontal gyrus (MFG) and right orbitofrontal cortex (OFC) was also observed in chronic tinnitus patients. In contrast, reduced directional connectivity was observed in the right cuneus in chronic tinnitus patients (Figures 2A,B and Table 2).

**TABLE 2:** Abnormal directional connectivity from the NAc to the other brain regions in tinnitus patients.

**Brain region**	**BA**	**MNI coordinates**	***T* score**	**Cluster size**
		***x*, *y*, *z* (mm)**		
**Seed: Left NAc**				
L inferior frontal gyrus	45	−54, 12, 24	2.8621	44
L cuneus	17	−9, −96, 0	−2.6429	41
**Seed: Right NAc**				
L middle frontal gyrus	10	−30, 60, 15	4.1236	59
R orbitofrontal cortex	11	42, 60, −12	3.3899	75
R cuneus	17	31, −87, 3	−3.0587	45

*Thresholds were set at a corrected *p* < 0.01, determined by Monte Carlo simulation. BA, Brodmann’s area; MNI, Montreal neurological institute; NAc, nucleus accumbens; cluster size is in cubic millimeters.*

### Directional Connectivity to the NAc

Compared with the controls, chronic tinnitus patients showed enhanced directional connection from the left middle temporal gyrus (MTG) and right MFG to the left NAc. In addition, in patients with tinnitus, the right MTG and the right IFG showed increased directional connectivity to the right NAc when compared with that in healthy controls (Figures 2C,D and Table 3). No reduction in directional connectivity with the NAc was observed.

**TABLE 3:** Abnormal directional connectivity from the other brain regions to the NAc in tinnitus patients.

**Brain region**	**BA**	**MNI coordinates**	***T* score**	**Cluster size**
		***x*, *y*, *z* (mm)**		
**Seed: Left NAc**				
R middle frontal gyrus	10	32, 46, 25	3.9302	65
R middle temporal gyrus	21	59, −53, 3	3.3662	75
**Seed: Right NAc**				
R inferior frontal gyrus	45	55, 21, 22	3.0863	51
R middle temporal gyrus	21	48, −60, 15	3.3322	93

*Thresholds were set at a corrected *p* < 0.01, determined by Monte Carlo simulation. BA, Brodmann’s area; MNI: Montreal neurological institute; NAc, nucleus accumbens; cluster size is in cubic millimeters.*

### Correlation Analysis

**FIGURE 3:**
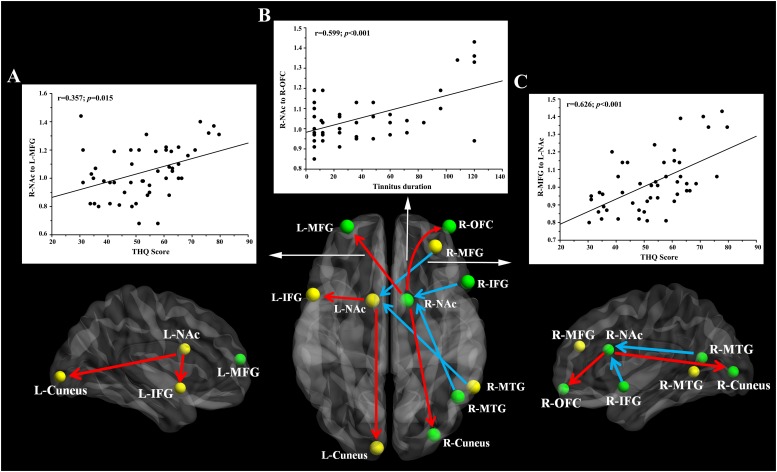
The directional functional connectivity networks of the bilateral NAc. The red line represents the directional functional connectivity from the bilateral NAc to the other brain regions; the blue line represents the directional functional connectivity from the other brain regions to the bilateral NAc. **(A)** Positive correlation between the increased directional connectivity from the right NAc to left MFG and the THQ score (*r* = 0.357, *p* = 0.015). **(B)** Positive correlation between the increased directional connectivity from the right NAc to right OFC and the tinnitus duration (*r* = 0.599, *p* < 0.001). **(C)** Positive correlation between the enhanced directional connectivity from the right MFG to left NAc and the THQ score (*r* = 0.626, *p* < 0.001).

Pearson’s correlation analyses revealed that THQ scores were positively correlated with the increased directional connectivity from the right NAc to the left MFG (*r* = 0.357, *p* = 0.015) and from the right MFG to the left NAc (*r* = 0.626, *p* < 0.001). In addition, Figure 3 shows a positive association between the enhanced directional connectivity from the right NAc to the right OFC and the tinnitus duration (*r* = 0.599, *p* < 0.001). These correlations were corrected for age, sex, education, and hearing thresholds. Other measures of increased or decreased directional connectivity were independent of tinnitus duration or THQ scores. None of the disrupted directional connectivity was correlated with SAS or SDS score (Table 4).

**TABLE 4:** Correlation coefficients and significance between the abnormal directional connectivity and tinnitus characteristics in tinnitus patients.

**Brain region**	**Tinnitus duration**	**THQ**	**SAS**	**SDS**
L-NAc to L-IFG	r = 0.108	r = 0.112	r = −0.149	r = 0.051
	p = 0.477	p = 0.401	p = 0.323	p = 0.736
L-NAc to L-cuneus	r = 0.180	r = 0.068	r = −0.276	r = −0.115
	p = 0.232	p = 0.652	p = 0.063	p = 0.446
R-NAc to L-MFG	r = 0.080	r = 0.357	r = 0.132	r = −0.051
	p = 0.597	p = 0.015^∗^	p = 0.384	p = 0.712
R-NAc to R-OFC	r = 0.599	r = 0.118	r = −0.025	r = 0.007
	p < 0.001^∗^	p = 0.435	p = 0.867	p = 0.963
R-MFG to L-NAc	r = 0.099	r = 0.626	r = −0.032	r = −0.051
	p = 0.514	p < 0.001^∗^	p = 0.834	p = 0.735
R-MTG to L-NAc	r = 0.245	r = −0.030	r = −0.026	r = −0.184
	p = 0.119	p = 0.841	p = 0.862	p = 0.221
R-MFG to R-NAc	r = 0.225	r = −0.013	r = 0.029	r = −0.158
	p = 0.128	p = 0.932	p = 0.851	p = 0.293
R-MTG to R-NAc	r = 0.205	r = −0.138	r = 0.064	r = −0.134
	p = 0.171	p = 0.360	p = 0.672	p = 0.375

*^∗^*p* < 0.05. THQ, tinnitus handicap questionnaires; SAS, self-rating anxiety scale; SDS, self-rating depression scale; NAc, nucleus accumbens; IFG, inferior frontal gyrus; MFG, middle frontal gyrus; OFC, orbitofrontal cortex; MTG, middle temporal gyrus; L, left; R, right.*

## Discussion

In this study, resting-state fMRI combined with GCA was used to investigate the directional connectivity between the NAc and all brain regions in chronic tinnitus patients compared with healthy controls. NAc was selected as the seed region of the brain networks, as it plays an important role in establishing the significant directional connectivity patterns in the study of tinnitus. Hullfish et al. (2019) studied whether the NAc and the extended frontal striatum network participate in the pathological process of tinnitus by using resting-state fMRI. Moreover, NAc is also implicated in the pathologies of several other disorders, namely, reward deficiency syndromes, including addiction, attention deficit hyperactivity disorder, and schizophrenia (Blum et al., 2000). However, no study has observed the altered directional functional connectivity from this core region in the frontostriatal network. We note that our approach of GCA is not a conceptual innovation but is an effective solution for investigating altered directional connectivity. Our fMRI study is the first to refer to this method and settle on the NAc as the seed region of tinnitus. To provide evidence for NAc-related neural network intercorrelations and particularly emphasize the core role of the NAc and the vmPFC in tinnitus neural circuits, specifically interactions in the frontostriatal gating system, we will discuss our results combined with those of the current literature (Rauschecker et al., 2015).

Reductions in GM volumes in the NAc, vmPFC, superior frontal gyrus, superior temporal gyrus, cingulate cortex, hippocampus, and occipital lobe in tinnitus patients have been reported by previous studies (Muhlau et al., 2006; Schneider et al., 2009; Leaver et al., 2011, 2012; Boyen et al., 2013; Adjamian et al., 2014). However, we did not detect any significant differences of GM volume between our tinnitus patients and controls. It is possible that the absence of any hearing loss in our tinnitus population may be one reason for the different results. In addition, the magnetic resonance (MR) technique and analytical method should also be taken into account.

Prior studies found that the NAc receives excitatory input from the neocortex, which ends on GABAergic spiny projection neurons directly and via inhibitory interneurons (Tepper et al., 2004). Moreover, a direct GABAergic projection from the basal ganglia back to the frontal cortex can be also detected (Saunders et al., 2015). In addition, the NAc receives modulatory input from dopaminergic structures. Dopaminergic activity has been linked to valuation, motivation, and learning in the NAc (Salamone and Correa, 2012; Guitart-Masip et al., 2014). It has therefore been claimed that dopamine and the NAc particularly relate to the valuation of tinnitus, which may influence the development of tinnitus into a chronic condition. We hypothesize that enhanced directional connectivity of NAc may increase the dopamine activity that will maintain the tinnitus perception.

Previous studies on tinnitus have provided evidence for decreased functional connectivity in the default mode network (DMN) (Husain and Schmidt, 2014; Chen et al., 2015, 2018a; Hinkley et al., 2015; Schmidt et al., 2017). In addition, in patients with chronic tinnitus, abnormal functional connectivity in limbic–auditory circuits plays a pivotal role in pathogenesis (Rauschecker et al., 2010; Leaver et al., 2011; Gunbey et al., 2017). More specifically, the subcallosal area in the limbic system is composed of the NAc and extends anteriorly toward the vmPFC (Rauschecker et al., 2010; Leaver et al., 2011; Middleton and Tzounopoulos, 2012; Adjamian et al., 2014; Carpenter-Thompson et al., 2014; De Ridder et al., 2014; Hinkley et al., 2015; Meyer et al., 2016; Hullfish et al., 2019). Therefore, Rauschecker et al. (2010) proposed a model in which the NAc and vmPFC participated in the elimination of the tinnitus signal at the level of the thalamus. Our results regarding NAc bidirectional functional connectivity were consistent with the outcomes above. The hyperactivity in the NAc and altered auditory–limbic networks may be responsible for the increased interactions between the NAc and auditory regions (Leaver et al., 2011; Adjamian et al., 2014; Rauschecker et al., 2015). Schmidt et al. (2017) found decreased DMN–precuneus connectivity and proposed correlations between interrupted connectivity and chronic tinnitus as well as tinnitus severity, which is in accordance with our result that there was decreased effective connectivity from the NAc to the bilateral cuneus. In addition, it was found that tinnitus distress was positively correlated with the increased effect of the NAc on the bilateral connection with the MFG and that the effective connection between the NAc and the OFC and MFG was enhanced. The overlapping regions of the MFG and OFC all include Brodmann’s area 10, which specifically refers to the vmPFC, reflecting an interwoven relationship with the NAc in the brain network of tinnitus patients.

It is noteworthy that there were strong interactions between the NAc and vmPFC regions in tinnitus. Previous studies noted that a dysfunctional frontostriatal system can be attributed to the anomalous structure and function of the NAc and vmPFC in tinnitus patients (Rauschecker et al., 2015; Leaver et al., 2016a; Sedley et al., 2016; Hullfish et al., 2019; Tzounopoulos et al., 2019). Moreover, GM volume reduction was also detected in the subcallosal area of the vmPFC in tinnitus patients (Muhlau et al., 2006; Leaver et al., 2011; Rauschecker et al., 2015). In addition, several studies demonstrated that limbic brain regions in tinnitus are affected by functional plasticity (Lockwood et al., 1998; Mirz et al., 2000; Plewnia et al., 2007). Complementing these findings, an event-related fMRI study was the first to reveal the stimulus-evoked hyperactivity of the NAc in tinnitus patients (Leaver et al., 2011). The sgACC/vmPFC region of highly distressed tinnitus patients showed specific functional connectivity that was related to tinnitus loudness (Vanneste et al., 2014). The latest resting-state fMRI study on tinnitus utilizing NAc as a seed region revealed that NAc-related connectivity was significantly reduced, while frontostriatal connections, such as NAc–vmPFC, were most likely negatively correlated with age and mean hearing loss (Hullfish et al., 2019). Accordingly, the significantly altered effective connectivity of NAc was observed, providing effective support for the previous hypothesis of NAc–vmPFC interactions.

Beyond the discussions mentioned above, converging evidence has shown that tinnitus, as a relatively circumscribed condition, may facilitate a better understanding of the common mechanisms in limbic dysregulations of many similar disorders (Leaver et al., 2011). An analogous study was conducted, since tinnitus and chronic pain have a high degree of similarity not only in terms of sensory perception disorder and the influence of behavior but also in their pathophysiological dysfunction mechanism in the neural hierarchy (Apkarian et al., 2011; Rauschecker et al., 2015). fMRI studies on chronic pain also showed structural and functional abnormities (May, 2011; Wasan et al., 2011; Howard et al., 2012; Liu et al., 2013; Smallwood et al., 2013; Cauda et al., 2014). In addition, Rauschecker et al. (2010, 2015) concluded that chronic pain showed obvious similarity in part of the top-down modulation of sensory signals, as well as the evaluation process, and therefore proposed that the vmPFC and the NAc are crucial in the frontostriatal gating mechanism in both sensory modalities, which is consistent with our result showing bilateral altered directional connections. The intrinsic NAc–vmPFC–TRN noise cancelation system was considered the formation mechanism of tinnitus (Rauschecker et al., 2010; Sedley et al., 2016). The inhibitory signal from the NAc–vmPFC system to the TRN decreases when it becomes abnormal. Once the noise-canceling system becomes inefficient or invalid, the extra noise will stop (Rauschecker et al., 2010). Increased directional connectivity from the NAc to the IFG and MFG was observed in our study. Aberrant intrinsic connections seem to exist in frontostriatal thalamic circuits, although there is no evidence in our results indicating direct decreased connectivity to the TRN in the thalamus. Moreover, increased connectivity instead of decreased connectivity has been found in regard to the altered directional connectivity to the NAc. Based on our results and the noise-canceling mechanism, we hypothesize that with a reduced inhibitory signal, the MGN-TRN not only fails to cancel the phantom sound but also feeds back more signal to the NAc through the amygdala, which may explain the increased directional connectivity from the NAc.

Several limitations must be acknowledged in this study. First, our ability to detect the relationship between abnormal directional connectivity and tinnitus characteristics may be reduced because of our small sample size. Additional longitudinal studies involving more subjects are required in the future. Furthermore, only the NAc was selected as the seed region to investigate the directional connectivity in tinnitus. For initial consideration, this study just would like to explore more deeply about the NAc-related connectivity in tinnitus and see whether any directional NAc-related connectivity has correlation with tinnitus-related distress, based on the study of Hullfish et al. (2019). Therefore, other critical seed regions were not included in our directional connectivity analysis. We admit that the selection of the seed region is not data driven, which will result in a biased conclusion. Other data-driven methods, such as independent component analysis (ICA) and principal component analysis (PCA), will be used for generating the seed regions in our future study. The current GCA approach can be extended to other regions within the frontostriatal gating circuit, such as the sgACC. Previous structural MRI and fMRI studies have suggested the critical role of the sgACC in tinnitus patients (Rauschecker et al., 2015; Meyer et al., 2016; Hullfish et al., 2019). Disruption of the descending projection from sgACC to the TRN might prevent the suppression of irrelevant sensory signals, such as those that result in a phantom percept, which might raise the intensity of such percepts (Rauschecker et al., 2015). Our prior study has also identified the relationship between tinnitus distress and abnormal functional connectivity in the ACC, especially the dorsal and ventral ACC (Chen et al., 2018b). However, the role of sgACC has not been demonstrated in this study. In addition, the NAc–sgACC interaction involved in the frontostriatal gating circuit has not been confirmed in the study of Hullfish et al. (2019). Therefore, the exact role of the sgACC on the neuropathological mechanism underlying chronic tinnitus will require exploration in further studies. Moreover, the subjects in the current study showed no obvious hearing loss or hyperacusis, which is not representative of all tinnitus patients. Finally, the auditory pathway is likely to be activated by scanner noise, which is nearly impossible to completely eliminate even with earplugs or active noise reduction (Rondinoni et al., 2013). This uncertainty should be taken into consideration when interpreting the resting-state fMRI data in future research.

## Conclusion

To our knowledge, this is the first fMRI study to apply the GCA method to explore the directional connectivity of the NAc in chronic tinnitus patients. The current study showed that tinnitus distress correlates with bidirectionally enhanced effective connectivity between the NAc and the prefrontal cortex. Taken together, these results help elucidate the neural basis of frontostriatal gating control of tinnitus sensation, which may play a pivotal role in the neuropathological features of tinnitus.

## Data Availability Statement

The datasets generated for this study are available on request to the corresponding author.

## Ethics Statement

The studies involving human participants were reviewed and approved by the Ethics Committee of Nanjing Medical University. The patients/participants provided their written informed consent to participate in this study.

## Author Contributions

J-JX and JC designed the experiment, collected the data, performed the analysis, and wrote the manuscript. YF, WY, HC, and YW helped to collect the data and perform the analysis. Y-CC and XY contributed to the discussion and manuscript revision.

## Conflict of Interest

The authors declare that the research was conducted in the absence of any commercial or financial relationships that could be construed as a potential conflict of interest.

## Footnotes

1 http://www.ansir.wfubmc.edu

2 http://www.fil.ion.ucl.ac.uk/spm

3 https://afni.nimh.nih.gov/pub/dist/doc/program_help/3dClustSim.html

## References

[B1] Adjamian P. Hall D. A. Palmer A. R. Allan T. W. Langers D. R. (2014). Neuroanatomical abnormalities in chronic tinnitus in the human brain. *Neurosci. Biobehav. Rev.* 45 119–133. 10.1016/j.neubiorev.2014.05.013 24892904 PMC4148481

[B2] Apkarian A. V. Hashmi J. A. Baliki M. N. (2011). Pain and the brain: specificity and plasticity of the brain in clinical chronic pain. *Pain* 152(3 Suppl.), S49–S64. 10.1016/j.pain.2010.11.010 21146929 PMC3045648

[B3] Baguley D. McFerran D. Hall D. (2013). Tinnitus. *Lancet* 382 1600–1607. 10.1016/s0140-6736(13)60142-60147 23827090

[B4] Barkhof F. Haller S. Rombouts S. A. R. B. (2014). Resting-state functional MR imaging:a new window to the brain. *Radiology* 272 29–49. 10.1148/radiol.14132388 24956047

[B5] Barry K. M. Paolini A. G. Robertson D. Mulders W. H. (2015). Modulation of medial geniculate nucleus neuronal activity by electrical stimulation of the nucleus accumbens. *Neuroscience* 308 1–10. 10.1016/j.neuroscience.2015.09.008 26349008

[B6] Bauer C. A. (2018). Tinnitus. *N. Engl. J. Med.* 378 1224–1231. 10.1056/NEJMcp1506631 29601255

[B7] Biswal B. Yetkin F. Z. Haughton V. M. Hyde J. S. (1995). Functional connectivity in the motor cortex of resting human brain using Echo-PlanarMRI. *Magn. Reson. Med.* 34 537–541. 10.1002/mrm.1910340409 8524021

[B8] Blum K. Braverman E. R. Holder J. M. Lubar J. F. Monastra V. J. Miller D. (2000). The reward deficiency syndrome: a biogenetic model for the diagnosis and treatment of impulsive, addictive and compulsive behaviors. *J. Psychol. Drugs* 32(Suppl.1), 1–112. 10.1080/02791072.2000.10736099 11280926

[B9] Boyen K. Langers D. R. de Kleine E. van Dijk P. (2013). Gray matter in the brain: differences associated with tinnitus and hearing loss. *Hear. Res.* 295 67–78. 10.1016/j.heares.2012.02.010 22446179

[B10] Carpenter-Thompson J. R. Akrofi K. Schmidt S. A. Dolcos F. Husain F. T. (2014). Alterations of the emotional processing system may underlie preserved rapid reaction time in tinnitus. *Brain Res.* 1567 28–41. 10.1016/j.brainres.2014.04.024 24769166

[B11] Cauda F. Palermo S. Costa T. Torta R. Duca S. Vercelli U. (2014). Gray matter alterations in chronic pain: a network-oriented meta-analytic approach. *Neuroimage Clin.* 4 676–686. 10.1016/j.nicl.2014.04.007 24936419 PMC4053643

[B12] Chen G.-D. Radziwon K. E. Kashanian N. Manohar S. Salvi R. (2014). Salicylate-induced auditory perceptual disorders and plastic changes in nonclassical auditory centers in rats. *Neural. Plast.* 2014 1–18. 10.1155/2014/658741 24891959 PMC4033555

[B13] Chen Y.-C. Feng Y. Xu J.-J. Mao C.-N. Xia W. Ren J. (2016). Disrupted brain functional network architecture in chronic tinnitus patients. *Front. Aging Neurosci.* 8:174 10.3389/fnagi.2016.00174 PMC4937025 27458377

[B14] Chen Y.-C. Liu S. Lv H. Bo F. Feng Y. Chen H. (2018b). Abnormal resting-state functional connectivity of the anterior cingulate cortex in unilateral chronic tinnitus patients. *Front. Neurosci.* 12:9. 10.3389/fnins.2018.00009 29410609 PMC5787069

[B15] Chen Y. C. Chen H. Bo F. Xu J. J. Deng Y. Lv H. (2018a). Tinnitus distress is associated with enhanced resting-state functional connectivity within the default mode network. *Neuropsychiatr. Dis. Treat* 14 1919–1927. 10.2147/NDT.S164619 30122924 PMC6078076

[B16] Chen Y. C. Xia W. Luo B. Muthaiah V. P. Xiong Z. Zhang J. (2015). Frequency-specific alternations in the amplitude of low-frequency fluctuations in chronic tinnitus. *Front. Neural. Circ.* 9:67. 10.3389/fncir.2015.00067 26578894 PMC4624866

[B17] Chen Y.-C. Yong W. Xing C. Feng Y. Haidari N. A. Xu J.-J. (2019). Directed functional connectivity of the hippocampus in patients with presbycusis. *Brain Imag. Behav.* 10.1007/s11682-019-00162-z [Epub ahead of print]. 31270776

[B18] Crippaa A. Lanting C. P. Dijk P. V. Roerdink J. B. T. M. (2010). A diffusion tensor imaging study on the auditory system and tinnitus. *Open Neuroimag. J.* 4 16–25. 10.2174/1874440001004010016 20922048 PMC2948149

[B19] De Ridder D. Vanneste S. Weisz N. Londero A. Schlee W. Elgoyhen A. B. (2014). An integrative model of auditory phantom perception: tinnitus as a unified percept of interacting separable subnetworks. *Neurosci. Biobehav. Rev.* 44 16–32. 10.1016/j.neubiorev.2013.03.021 23597755

[B20] Eggermont J. J. (2007). Correlated neural activity as the driving force for functional changes in auditory cortex. *Hear. Res.* 229 69–80. 10.1016/j.heares.2007.01.008 17296278

[B21] Eggermont J. J. Roberts L. E. (2004). The neuroscience of tinnitus. *Trends Neurosci.* 27 676–682. 10.1016/j.tins.2004.08.010 15474168

[B22] Galazyuk A. V. Wenstrup J. J. Hamid M. A. (2012). Tinnitus and underlying brain mechanisms. *Curr. Opin. Otolaryngol. Head. Neck. Surg.* 20 409–415. 10.1097/MOO.0b013e3283577b81 22931904 PMC3886369

[B23] Good C. D. Johnsrude I. S. Ashburner J. Henson R. N. Friston K. J. Frackowiak R. S. (2001). A voxel-based morphometric study of ageing in 465 normal adult human brains. *Neuroimage* 14(1 Pt 1), 21–36. 10.1006/nimg.2001.0786 11525331

[B24] Guitart-Masip M. Duzel E. Dolan R. Dayan P. (2014). Action versus valence in decision making. *Trends Cogn. Sci.* 18 194–202. 10.1016/j.tics.2014.01.003 24581556 PMC3989998

[B25] Gunbey H. P. Gunbey E. Aslan K. Bulut T. Unal A. Incesu L. (2017). Limbic-auditory interactions of tinnitus: an evaluation using diffusion tensor imaging. *Clin. Neuroradiol.* 27 221–230. 10.1007/s00062-015-0473-470 26490370

[B26] Guo W. Liu F. Zhang Z. Liu J. Yu M. Zhang J. (2015). Unidirectionally affected causal connectivity of cortico-limbic-cerebellar circuit by structural deficits in drug-naive major depressive disorder. *J. Affect. Disord.* 172 410–416. 10.1016/j.jad.2014.10.019 25451445

[B27] Hinkley L. B. Mizuiri D. Hong O. Nagarajan S. S. Cheung S. W. (2015). Increased striatal functional connectivity with auditory cortex in tinnitus. *Front. Hum. Neurosci.* 9:568. 10.3389/fnhum.2015.00568 26578924 PMC4623204

[B28] Howard M. A. Sanders D. Krause K. O’Muircheartaigh J. Fotopoulou A. Zelaya F. (2012). Alterations in resting-state regional cerebral blood flow demonstrate ongoing pain in osteoarthritis: an arterial spin-labeled magnetic resonance imaging study. *Arth. Rheum.* 64 3936–3946. 10.1002/art.37685 22933378

[B29] Hullfish J. Abenes I. Yoo H. B. De Ridder D. Vanneste S. (2019). Frontostriatal network dysfunction as a domain-general mechanism underlying phantom perception. *Hum. Brain Mapp.* 40 2241–2251. 10.1002/hbm.24521 30648324 PMC6865744

[B30] Husain F. T. Schmidt S. A. (2014). Using resting state functional connectivity to unravel networks of tinnitus. *Hear. Res.* 307 153–162. 10.1016/j.heares.2013.07.010 23895873

[B31] Jiao Q. Lu G. Zhang Z. Zhong Y. Wang Z. Guo Y. (2011). Granger causal influence predicts BOLD activity levels in the default mode network. *Hum. Brain Mapp.* 32 154–161. 10.1002/hbm.21065 21157880 PMC6870036

[B32] Khalfa S. Dubal S. Veuillet E. Perez-Diaz F. Jouvent R. Collet L. (2002). Psychometric normalization of a hyperacusis questionnaire. *ORL J. Otorhinolaryngol. Relat. Spec.* 64 436–442. 10.1159/000067570 12499770

[B33] Kim J. Y. Kim Y. H. Lee S. Seo J. H. Song H. J. Cho J. H. (2012). Alteration of functional connectivity in tinnitus brain revealed by resting-state fMRI? A pilot study. *Int. J. Audiol.* 51 413–417. 10.3109/14992027.2011.652677 22283490

[B34] Knipper M. Van Dijk P. Nunes I. Ruttiger L. Zimmermann U. (2013). Advances in the neurobiology of hearing disorders: recent developments regarding the basis of tinnitus and hyperacusis. *Prog. Neurobiol.* 111 17–33. 10.1016/j.pneurobio.2013.08.002 24012803

[B35] Kreuzer P. M. Lehner A. Schlee W. Vielsmeier V. Schecklmann M. Poeppl T. B. (2015). Combined rTMS treatment targeting the anterior cingulate and the temporal cortex for the treatment of chronic tinnitus. *Sci. Rep.* 5:18028. 10.1038/srep18028 26667790 PMC4678896

[B36] Kuk F. K. Tyler R. S. Russell D. Jordan H. (1990). The psychometric properties of a tinnitus handicap questionnaire. *Ear Hear.* 11 434–445. 10.1097/00003446-199012000-00005 2073977

[B37] Langers D. R. de Kleine E. van Dijk P. (2012). Tinnitus does not require macroscopic tonotopic map reorganization. *Front. Syst. Neurosci.* 6:2 10.3389/fnsys.2012.00002 PMC3269775 22347171

[B38] Leaver A. M. Renier L. Chevillet M. A. Morgan S. Kim H. J. Rauschecker J. P. (2011). Dysregulation of limbic and auditory networks in tinnitus. *Neuron* 69 33–43. 10.1016/j.neuron.2010.12.002 21220097 PMC3092532

[B39] Leaver A. M. Seydell-Greenwald A. Rauschecker J. P. (2016a). Auditory-limbic interactions in chronic tinnitus: challenges for neuroimaging research. *Hear. Res.* 334 49–57. 10.1016/j.heares.2015.08.005 26299843 PMC7343340

[B40] Leaver A. M. Turesky T. K. Seydell-Greenwald A. Morgan S. Kim H. J. Rauschecker J. P. (2016b). Intrinsic network activity in tinnitus investigated using functional MRI. *Hum. Brain Mapp.* 37 2717–2735. 10.1002/hbm.23204 27091485 PMC4945432

[B41] Leaver A. M. Seydell-Greenwald A. Turesky T. K. Morgan S. Kim H. J. Rauschecker J. P. (2012). Cortico-limbic morphology separates tinnitus from tinnitus distress. *Front. Syst. Neurosci.* 6:21. 10.3389/fnsys.2012.00021 22493571 PMC3319920

[B42] Liu J. Hao Y. Du M. Wang X. Zhang J. Manor B. (2013). Quantitative cerebral blood flow mapping and functional connectivity of postherpetic neuralgia pain: a perfusion fMRI study. *Pain* 154 110–118. 10.1016/j.pain.2012.09.016 23140909

[B43] Lockwood A. H. Salvi R. J. Coad M. L. Towsley M. L. Wack D. S. Murphy B. W. (1998). The functional neuroanatcomy of tinnitus evidence for limbic system links arid neural plasticity. *Neurology* 50 114–120. 10.1212/wnl.50.1.114 9443467

[B44] Lopez-Escamez J. A. Carey J. Chung W. H. Goebel J. A. Magnusson M. Mandala M. (2015). Diagnostic criteria for Meniere’s disease. *J. Vestib. Res.* 25 1–7. 10.3233/ves-150549 25882471

[B45] May A. (2011). Structural brain imaging: a window into chronic pain. *Neuroscientist* 17 209–220. 10.1177/1073858410396220 21489967

[B46] Meyer M. Neff P. Liem F. Kleinjung T. Weidt S. Langguth B. (2016). Differential tinnitus-related neuroplastic alterations of cortical thickness and surface area. *Hear. Res.* 342 1–12. 10.1016/j.heares.2016.08.016 27671157

[B47] Middleton J. W. Tzounopoulos T. (2012). Imaging the neural correlates of tinnitus: a comparison between animal models and human studies. *Front. Syst. Neurosci.* 6:35. 10.3389/fnsys.2012.00035 22586378 PMC3343475

[B48] Mirz F. Gjeddek A. Ishizu O. Pedersen C. B. (2000). Cortical networks subserving the perception of tinnitus—a PET study. *Acta Otolaryngol. Suppl.* 543 241–243. 10.1080/000164800454503 10909031

[B49] Muhlau M. Rauschecker J. P. Oestreicher E. Gaser C. Rottinger M. Wohlschlager A. M. (2006). Structural brain changes in tinnitus. *Cereb. Cortex* 16 1283–1288. 10.1093/cercor/bhj070 16280464

[B50] Plewnia C. Reimold M. Najib A. Brehm B. Reischl G. Plontke S. K. (2007). Dose-dependent attenuation of auditory phantom perception (tinnitus) by PET-guided repetitive transcranial magnetic stimulation. *Hum. Brain Mapp.* 28 238–246. 10.1002/hbm.20270 16773635 PMC6871343

[B51] Raichle M. E. MacLeod A. M. Snyder A. Z. Powers W. J. Gusnard D. A. (2000). a default mode of brain function. *Proc Natl. Acad. Sci. U.S.A.* 98 676–682. 10.1073/pnas.98.2.676 PMC14647 11209064

[B52] Rauschecker J. P. Leaver A. M. Mühlau M. (2010). Tuning out the noise Limbic-auditory interactions in tinnitus. *Neuron* 66 819–826. 10.1016/j.neuron.2010.04.032 20620868 PMC2904345

[B53] Rauschecker J. P. May E. S. Maudoux A. Ploner M. (2015). Frontostriatal gating of tinnitus and chronic pain. *Trends Cogn. Sci.* 19 567–578. 10.1016/j.tics.2015.08.002 26412095 PMC4587397

[B54] Rondinoni C. Amaro E. Jr. Cendes F. Dos Santos A. Salmon C. (2013). Effect of scanner acoustic background noise on strict resting-state fMRI. *Braz. J. Med. Biol. Res.* 46 359–367. 10.1590/1414-431x20132799 23579634 PMC3854411

[B55] Salamone J. D. Correa M. (2012). The mysterious motivational functions of mesolimbic dopamine. *Neuron* 76 470–485. 10.1016/j.neuron.2012.10.021 23141060 PMC4450094

[B56] Saunders A. Oldenburg I. A. Berezovskii V. K. Johnson C. A. Kingery N. D. Elliott H. L. (2015). A direct GABAergic output from the basal ganglia to frontal cortex. *Nature* 521:85. 10.1038/nature14179 25739505 PMC4425585

[B57] Schmidt S. A. Carpenter-Thompson J. Husain F. T. (2017). Connectivity of precuneus to the default mode and dorsal attention networks: a possible invariant marker of long-term tinnitus. *Neuroimage Clin.* 16 196–204. 10.1016/j.nicl.2017.07.015 28794980 PMC5542421

[B58] Schneider P. Andermann M. Wengenroth M. Goebel R. Flor H. Rupp A. (2009). Reduced volume of Heschl’s gyrus in tinnitus. *Neuroimage* 45 927–939. 10.1016/j.neuroimage.2008.12.045 19168138

[B59] Sedley W. Friston K. J. Gander P. E. Kumar S. Griffiths T. D. (2016). An integrative tinnitus model based on sensory precision. *Trends Neurosci.* 39 799–812. 10.1016/j.tins.2016.10.004 27871729 PMC5152595

[B60] Shargorodsky J. Curhan G. C. Farwell W. R. (2010). Prevalence and characteristics of tinnitus among US adults. *Am. J. Med.* 123 711–718. 10.1016/j.amjmed.2010.02.015 20670725

[B61] Smallwood R. F. Laird A. R. Ramage A. E. Parkinson A. L. Lewis J. Clauw D. J. (2013). Structural brain anomalies and chronic pain: a quantitative meta-analysis of gray matter volume. *J. Pain* 14 663–675. 10.1016/j.jpain.2013.03.001 23685185 PMC4827858

[B62] Song J. J. De Ridder D. Van de Heyning P. Vanneste S. (2012). Mapping tinnitus-related brain activation: an activation-likelihood estimation metaanalysis of PET studies. *J. Nucl. Med.* 53 1550–1557. 10.2967/jnumed.112.102939 22917883

[B63] Song J. J. Vanneste S. De Ridder D. (2015). Dysfunctional noise cancelling of the rostral anterior cingulate cortex in tinnitus patients. *PLoS One* 10:e0123538. 10.1371/journal.pone.0123538 25875099 PMC4395158

[B64] Stephan K. E. Roebroeck A. (2012). A short history of causal modeling of fMRI data. *Neuroimage* 62 856–863. 10.1016/j.neuroimage.2012.01.034 22248576

[B65] Tepper J. M. Koós T. Wilson C. J. (2004). GABAergic microcircuits in the neostriatum. *Trends Neurosci.* 27 662–669. 10.1016/j.tins.2004.08.007 15474166

[B66] Tzounopoulos T. Balaban C. Zitelli L. Palmer C. (2019). Towards a mechanistic-driven precision medicine approach for tinnitus. *J. Assoc. Res. Otolaryngol.* 20 115–131. 10.1007/s10162-018-00709-709 30825037 PMC6453992

[B67] Vanneste S. Congedo M. De Ridder D. (2014). Pinpointing a highly specific pathological functional connection that turns phantom sound into distress. *Cereb. Cortex* 24 2268–2282. 10.1093/cercor/bht068 23632885

[B68] Vanneste S. De Ridder D. (2012). The auditory and non-auditory brain areas involved in tinnitus. an emergent property of multiple parallel overlapping subnetworks. *Front. Syst. Neurosci.* 6:31. 10.3389/fnsys.2012.00031 22586375 PMC3347475

[B69] Wasan A. D. Loggia M. L. Chen L. Q. Napadow V. Kong J. Gollub R. L. (2011). Neural correlates of chronic low back pain measured by arterial spin labeling. *Anesthesiology* 115,364–374. 10.1097/ALN.0b013e318220e880 PMC3286828 21720241

[B70] Yan C.-G. Wang X.-D. Zuo X.-N. Zang Y.-F. (2016). DPABI: data processing & analysis for (resting-state) brain imaging. *Neuroinformatics* 14 339–351. 10.1007/s12021-016-9299-4 27075850

[B71] Yu E. Liao Z. Mao D. Zhang Q. Ji G. Li Y. (2017). Directed functional connectivity of posterior cingulate cortex and whole brain in Alzheimer’s disease and mild cognitive impairment. *Curr. Alzheimer Res.* 14 628–635. 10.2174/1567205013666161201201000 27915993

[B72] Zang Z.-X. Yan C.-G. Dong Z.-Y. Huang J. Zang Y.-F. (2012). Granger causality analysis implementation on MATLAB: a graphic user interface toolkit for fMRI data processing. *J. Neurosci. Methods* 203 418–426. 10.1016/j.jneumeth.2011.10.006 22020117

[B73] Zhong Y. Huang L. Cai S. Zhang Y. von Deneen K. M. Ren A. (2014). Altered effective connectivity patterns of the default mode network in Alzheimer’s disease: an fMRI study. *Neurosci. Lett.* 578 171–175. 10.1016/j.neulet.2014.06.043 24996191 PMC6293460

[B74] Zung W. (1986). “Zung self-rating depression scale and depression status inventory,” in *Assessment of Depression*, eds Sartorius N. Ban T. A. (Berlin: Springer), 221–231. 10.1007/978-3-642-70486-4_21

[B75] Zung W. W. (1971). A rating instrument for anxiety disorders. *Psychosomatics* 12 371–379. 10.1016/s0033-3182(71)71479-0 5172928

